# Molecular Mechanisms and Biomarkers Associated with Chemotherapy-Induced AKI

**DOI:** 10.3390/ijms23052638

**Published:** 2022-02-27

**Authors:** Letizia De Chiara, Gianmarco Lugli, Gianluca Villa, Valentina Raglianti, Faeq Husain-Syed, Fiammetta Ravaglia, Paola Romagnani, Elena Lazzeri

**Affiliations:** 1 Department of Experimental and Clinical Biomedical Sciences “Mario Serio”, University of Florence, 50139 Florence, Italy; gianmarco.lugli@unifi.it (G.L.); valentinaraglianti@gmail.com (V.R.); paola.romagnani@unifi.it (P.R.); 2 Nephrology and Dialysis Unit, Meyer Children’s Hospital, 50139 Florence, Italy; 3 Department of Health Sciences, Section of Anesthesiology, Intensive Care and Pain Medicine, University of Florence, 50134 Florence, Italy; gianluca.villa@unifi.it; 4 Department of Internal Medicine II, University Hospital Giessen and Marburg, Justus-Liebig-University Giessen, 35392 Giessen, Germany; faeq.husain-syed@innere.med.uni-giessen.de; 5 Nephrology and Dialysis Unit, Santo Stefano Hospital, 59100 Prato, Italy; ravaglia.fiammetta@gmail.com

**Keywords:** AKI, chemotherapy, biomarkers, polyploidy, nephrotoxicity, CKD

## Abstract

Acute kidney injury (AKI) is a life-threatening condition characterized by a rapid and transient decrease in kidney function. AKI is part of an array of conditions collectively defined as acute kidney diseases (AKD). In AKD, persistent kidney damage and dysfunction lead to chronic kidney disease (CKD) over time. A variety of insults can trigger AKI; however, chemotherapy-associated nephrotoxicity is increasingly recognized as a significant side effect of chemotherapy. New biomarkers are urgently needed to identify patients at high risk of developing chemotherapy-associated nephrotoxicity and subsequent AKI. However, a lack of understanding of cellular mechanisms that trigger chemotherapy-related nephrotoxicity has hindered the identification of effective biomarkers to date. In this review, we aim to (1) describe the known and potential mechanisms related to chemotherapy-induced AKI; (2) summarize the available biomarkers for early AKI detection, and (3) raise awareness of chemotherapy-induced AKI.

## 1. Introduction

Acute kidney injury (AKI) is a life-threatening condition with increasing incidence worldwide [1]. It is characterized by a rapid and transient decrease in kidney function, measured as an increase in serum creatinine (sCr) and a reduction in the glomerular filtration rate (GFR) and urine output [1]. AKI is part of an assortment of conditions, defined as acute kidney diseases (AKD) [1]. AKD can occur after an AKI event has ended, but where the deterioration in kidney dysfunction and structural damage persist or when kidney dysfunction evolves slowly without a rapid AKI onset [1]. AKD lasting for >3 months is referred to as chronic kidney disease (CKD) [2]. Approximately 20% to 50% of all patients admitted to the intensive care unit (ICU) develop AKI [3]. In the context of cancer treatment, old and new chemotherapy drugs often cause chemotherapy-associated nephrotoxicity [4,5]; accordingly, up to 17.5% of cancer patients develop AKI, which negatively affects patient survival [6]. Additionally, long-term, these AKI events are associated with the progression of CKD, cardiovascular complications, and mortality [7].

A common pathological feature of AKI is an injury to tubular epithelial cells (TEC), endothelial damage, and accumulation of inflammatory cells [1]. However, the incomplete understanding of pathophysiology and molecular mechanisms associated with chemotherapy-related nephrotoxicity resulting in AKI has hampered the identification of effective biomarkers for patient stratification. As chemotherapy-related AKI may affect the bioavailability of many chemotherapy drugs, potentially leading to suboptimal treatments, the identification of biomarker profiles predictive of AKI severity and outcome is now more urgent than ever. While routine diagnostic markers, such as sCr and urine output, measure the loss of kidney function following an AKI, they do not identify the preceding pathophysiological changes, such as tubular injury. Numerous urinary biomarkers are in use or have been proposed as indicators of kidney injury [8]. Identifying patients at high risk of developing AKI, together with an awareness of potential drug nephrotoxicity, early recognition, and management of incipient AKI, are vital to reducing cases of established AKI. In this review, we aim to (1) provide an overview of the mechanisms involved in chemotherapy-associated AKI; (2) discuss the biomarkers currently available and propose additional biomarkers for early AKI detection, and (3) raise awareness of chemotherapy-induced AKI and promote collaboration between nephrologists, oncologists, and intensive care specialists for early AKI recognition and effective oncologic patient management.

## 2. Epidemiology

According to incidence, prevalence, and mortality trends, the epidemiology of cancer worldwide is dramatically changing [9,10]. Despite the impact of cancer overdiagnosis in correctly estimating the actual impact of cancer on patient survival [11], cancer will likely be the leading cause of death in the following decades [9,10]. Nevertheless, the development of new cancer drugs continues to improve cancer survival rates in high-income countries [11]. Chemotherapy nephrotoxicity is associated with significant kidney manifestations, including AKI, progression to CKD, proteinuria, nephrotic syndrome, and electrolyte disorders [9]. Thus, the interplay between cancer treatment and kidney health is complex. While cancer patients are a population at high risk of developing AKI as a result of their cancer treatment regimes, the physical and psychosocial effects associated with cancer survivorship may make a subsequent CKD diagnosis a difficult health issue to address [12].

In recent years, many initiatives have provided operative definitions of AKI, all based on the measurement of sCr and urine output (Table 1). AKI can complicate disease courses and is associated with increased mortality in cancer patients [6]. In a population-based study of 163,071 patients undergoing systemic treatment for cancer in Ontario, the overall cumulative incidence of AKI was 9.3% [13]. A similar study conducted in China demonstrated an incidence of 7.5%, with a higher prevalence among hospitalized patients [14]. The most extensive study to date, accounting for 1.2 million patients in Denmark followed from 1999 to 2006, revealed a 1-year AKI risk of 17.5% [15]. Thus, risk factors for AKI, AKD, and progression to CKD should be carefully assessed [6].

The number of cancer patients admitted to the ICU has gradually increased over the last number of decades [16,17]. A recent multicenter, observational study showed that 15% of patients admitted to European ICUs have cancer, with solid tumors being more common than hematological cancer (85% vs. 15%, respectively) [18]. AKI is a severe and frequent complication during critical illness ranging from 54% [19] to 70% [20,21], and it is particularly common in patients with hematological cancer or multiple myeloma [18,22]. Notably, in most cases, AKI is already present on admission to ICU rather than acquired in ICU [21]. However, reports vary depending on the criteria used to define AKI [23]. Furthermore, diagnosis and staging of AKI in critically ill patients should consider that sCr concentrations may be artificially low due to cachexia and muscle mass loss, although AKI is already present.

## 3. Risk Factors

Epidemiology studies have highlighted common risk factors for AKI, also traceable in the general population, and specific risk factors which are tumor-related. In advanced cancer, underlying CKD and diabetes are all associated with an increased risk of AKI [13], together with urinary tract obstruction [14]. In addition, volume depletion, due to fluid loss or confinement into the third space, a common condition in elderly patients, can be easily traced as one of the most common risk factors for AKI [29]. Other drugs, when administered concomitantly with certain cancer drugs, such as diuretics, angiotensin-converting enzyme (ACE)-inhibitors, or proton-pump inhibitors, are associated with increased toxicity. Tumor-specific risk factors are generally a hallmark of certain tumors [30,31]. AKI complicating hematologic malignancies may be due to light chain cast nephropathy in multiple myeloma or tumor lysis syndrome after the initiation of chemotherapy in patients with high-grade lymphomas or leukemias [30,31]. Metastases to the kidney from solid tumors are not uncommon; functional impairment of the kidneys generally requires metastases to both kidneys. This condition occurs mainly with rapidly growing hematologic malignancies, such as lymphoma or acute leukemia [32]. Thrombotic microangiopathy may be associated with primary cancer or, more likely, with therapeutic regimens, such as gemcitabine or vascular endothelial growth factor (VEGF) inhibitors (such as bevacizumab) [33]. Urinary tract obstruction should be considered as a cause of AKI in cancer patients, especially those with malignancies of the bladder, prostate, uterus, or cervix [34]. Intratubular obstruction can be caused by crystals composed of uric acid, xanthine, hypoxanthine, or calcium phosphate [35]. Of note, phosphate metabolism itself is dysregulated during AKI, and hyperphosphatemia can be present as a result of reduced kidney excretion together with increased fibroblast growth factor 23 (FGF-23) levels [36,37]. FGF-23 is a crucial modulator of calcium and phosphate metabolism. In vitro, FGF-23 is overexpressed in osteoblast-like cells exposed to chemotherapeutics [38], while it has been established that FGF-23 may be upregulated in some cancers [39]. Together, this data indicates an interplay between FGF-23, AKI, and chemotherapy administration that should be further investigated. Extrarenal obstruction can be caused by a wide range of malignancies and may indicate metastatic disease [40]. A diagnosis is usually established by imaging studies which typically show hydronephrosis [41]. When dealing with oncology patients, tumor-specific risk factors, together with common risk factors, significantly increase the risk of AKI. Indeed, these patients should receive additional attention to reduce the burden of AKI and CKD.

In critically ill patients with cancer, sepsis, metabolic disturbances (e.g., hypercalcemia and hyperuricemia), and the nephrotoxic effects of anticancer and supportive therapies are common triggers of AKI [18,42,43]. Older age (>65 years), female sex, and coexisting disease processes, including CKD, diabetic kidney disease, and volume depletion (e.g., due to vomiting or diarrhea), or renal hypoperfusion (e.g., due to cardiomyopathy, cirrhosis, or the nephrotic syndrome), predispose these patients to potential AKI development [44]. The association between AKI and anticancer therapies seems particularly relevant in the critical care setting [45]. Hyperthermic chemotherapy is a valuable strategy for patients with carcinomatosis [46]. Most surgical patients treated with cytoreductive surgery and hyperthermic intraperitoneal chemotherapy are admitted to the ICU and frequently develop severe AKI [46,47]. Immune checkpoint inhibitors (ICPi), which is one of the most frequently prescribed anticancer treatments nowadays [48], are associated with a unique spectrum of immune-related adverse events affecting several organs, including the kidneys [49]. Direct renal toxicity of these drugs can have severe consequences and lead to ICU admission.

Collectively, a better understanding of the mechanisms linked to chemotherapy-related AKI could potentially help the identification of more specific and sensitive biomarkers.

## 4. Mechanisms and Clinical Manifestations

AKI can affect the different portions of the nephron, namely the tubules and the glomeruli, as well as the interstitium and the vasculature [50]. Acute tubular necrosis (ATN) results from direct injury to the tubules and is one of the common manifestations of nephrotoxic AKI [50]. ATN is a dynamic process involving different forms of regulated necrosis, resulting in synchronization of tubular cell death along the entire tubule [51,52]. Necrotic TEC release pro-inflammatory molecules that activate resident immune cells in the interstitium, which, in turn, further promote tubular necrosis in a vicious circle [53]. Following AKI, functional recovery occurs via two main mechanisms: (1) clonal expansion of a TEC subset (termed progenitor cells) endowed with regenerative ability to replace lost TEC [54] and (2) polyploidization of differentiated TEC [54]. From an evolutionary point of view, polyploidization appears to be most likely developed to sustain a temporary functional recovery of the kidney that is not accompanied by a structural recovery (which should be sustained by progenitor cells). When structural damage is prolonged, AKI may progress to AKD [1].

**Figure 1:**
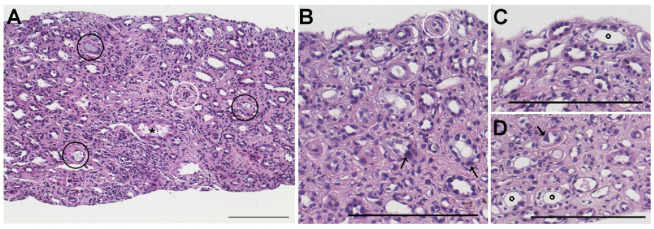
Chemotherapy-induced nephrotoxicity. (**A**) Hematoxylin and Eosin staining shows tubular damage in a kidney biopsy of a patient following treatment with a cocktail of cisplatin, carboplatin, etoposide, cyclophosphamide, and vincristine. (**B**–**D**) Higher magnification of biopsy shown in (**A**). Black circles indicate distal tubular casts. White circles indicate luminal cellular debris. * indicates proximal tubule injury. **°** indicates regenerative nuclear atypia. Arrows indicate karyomegaly. Bars 100 µm.

**Figure 2:**
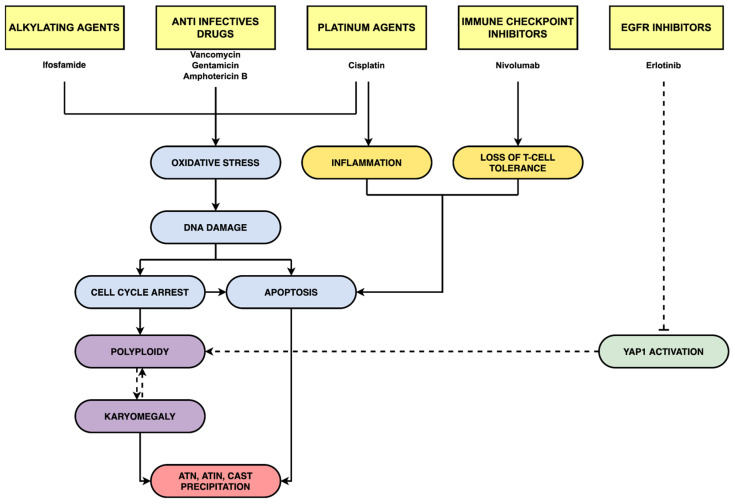
Demonstrated and putative chemotherapy-induced nephrotoxicity mechanisms in tubular epithelial cells. Schematic representation of the various mechanisms through which the drugs reported in this review cause nephrotoxicity. Dotted lines indicate putative mechanisms. ATN: Acute Tubular Necrosis; ATIN: Acute Tubulointerstitial Nephritis.

As a comprehensive analysis of all the AKI mechanisms associated with cancer is beyond the scope of this review, we will focus specifically on drugs that directly affect the tubules. Cytotoxic chemotherapy, targeted agents, as well as ICPi account for several cases of AKI in patients receiving those treatments. Nephrotoxicity is more frequently observed with cytotoxic agents, likely due to their nonspecific mechanisms of action [44]. Many of the drug-related mechanisms of nephrotoxicity are not well-defined, making it difficult to develop targeted strategies for preventing or minimizing their occurrence. In addition, there is often a lack of standardization for dose adjustment in patients with pre-existing kidney impairment [5]. The mechanisms of nephrotoxicity are summarized in Figure 1 and Figure 2, and Table 2 and described in the following sections.

*Cytotoxic agents*. There are many different classes of nephrotoxic agents employed for cancer treatment, comprising but not limited to alkylating agents, antimetabolites, antimicrotubule agents, antibiotics, proteasome inhibitors, and platinum agents. Among these, the most widely used (being prescribed in nearly 50% of all tumor chemotherapies [55]) is cisplatin, a platinum drug. Cisplatin nephrotoxicity may be associated with a protean clinical manifestation [56]. Appropriately timed renal functional assessment may help diagnose cisplatin-associated AKI, as exposure typically exerts a slow rise in sCr five to seven days after administration [56]. Severe AKI requiring kidney replacement therapy (KRT) is uncommon. Hypomagnesemia, a typical feature of cisplatin toxicity, is caused by urinary magnesium wasting, and it is dose-related [57,58]. 

Cisplatin-induced nephrotoxicity is associated with oxidative stress and inflammation [59,60,61]; however, the precise mechanisms of action of the drug remain somewhat unclear [59,62]. However, its major reported cytotoxic effect is mediated by its interaction with DNA, which leads to DNA damage and apoptosis induction [63]. Terminally differentiated cells, such as TEC, must cope with the accumulation of damage over the course of a lifespan [64]. Importantly, DNA damage triggered by cisplatin and the associated DNA damage response (DDR) is an important pathogenic mechanism of AKI following cisplatin treatment [65]. DDR activation may lead to cell cycle arrest [66,67,68] or, in the presence of severe injury, cell death. However, not only cell cycle arrest but also polyploidy has been recently shown to protect against DNA damage-induced cell death [64]. Cisplatin treatment in humans and rodents is reported to cause karyomegaly in renal tubules [69,70,71], which could indicate the presence of polyploid TEC. Indeed, renal tubule karyomegaly does not develop immediately, instead requiring successive rounds of nuclear division to increase the ploidy content to a recognizable size [72,73] and likely explaining why this feature is frequently missed.

Another commonly used nephrotoxic agent is ifosfamide, an alkylating agent. Its nephrotoxicity is particularly relevant considering that it is mostly observed in pediatric patients [74,75]. Thirty percent of the children treated with ifosfamide will consequently develop CKD [76]. Nevertheless, the reported prevalence of nephrotoxicity ranges from 15% to 60% [77,78]. Clinically, AKI associated with ifosfamide is characterized by tubular dysfunction [75]. In fact, ifosfamide mainly affects the S3 segment of the proximal tubule and/or the distal nephron resulting in Fanconi syndrome. This syndrome is characterized by inadequate reabsorption in the proximal renal tubules, with traceable glucosuria, aminoaciduria, tubular proteinuria, decreased phosphate reabsorption, and type 1 (distal) or type 2 (proximal) renal tubular acidosis, or even nephrogenic diabetes insipidus [75]. A specific risk factor for ifosfamide nephrotoxicity is cumulative drug dose [79,80]. Two drugs with antioxidants properties—mesna and N-acetylcysteine (NAC)—are currently used to limit its toxic effects, although their efficacy has not been tested in clinical trials [74,77,79,81]. Considering the long life expectancy of children and young adults surviving cancer, drug-related nephrotoxicity and its lasting consequences represent a crucial unmet problem in medicine. Among the numerous side effects associated with its metabolites [82,83,84], ifosfamide reacts with DNA molecules to form intra-and interstrand cross-links, causing the DNA strand to break [85]. Interestingly, ifosfamide has also been associated with karyomegalic nephropathy, further suggesting an interesting association between DNA damage, AKI, and increased ploidy [86,87,88]. Anti-infective drugs, such as vancomycin, gentamicin, and amphotericin B, are also leading causes of drug-induced nephrotoxicity [89,90,91]. Their mechanisms of action are not well understood, but their primary target is, in all cases, the proximal tubular cells where they cause oxidative stress [89,90,91], a well-recognized trigger of DNA damage [92]. Finally, one nephrotoxic manifestation of many cytotoxic agents is rhabdomyolysis [93,94,95] which is known to cause nephrotoxic AKI [96]. Importantly, we have recently shown that rhabdomyolysis triggers TEC polyploidy in response to damage [54].

Collectively, the presence of tubular karyomegaly and polyploidy in response to AKI may play an important role in the pathogenesis of nephrotoxicity, at least for some anti-cancer drugs.

Immune checkpoint inhibitors (ICPi). Immune checkpoint inhibitors (ICPi) are a major class of cancer drugs able to improve prognosis in several cancers. These humanized monoclonal antibodies target inhibitory receptors (CTLA-4, PD-1, LAG-3, TIM-3) and ligands (PD-L1) expressed on T lymphocytes, antigen-presenting cells, and tumor cells, eliciting an anti-tumor response by stimulating the immune system [97,98,99]. Targeting checkpoints of immune cell activation has been demonstrated to be the most effective approach for activating anti-tumor immune responses. The combination of CTLA-4 and PD-1 blockers increases the response rates in patients, and ipilimumab (anti-CTLA-4) plus nivolumab (anti-PD-1) in combination are particularly effective in different cancer types, such as those affecting the kidney [100].

However, patients treated with ICPi are also subject to “immune-related adverse events (IRAEs)”, which are common and can affect any organ, including lung, liver, skin, and kidney [101]. Recent data show differences in the IRAE characteristics caused by different ICPi, and organ-specific effects remain unexplained [102]. ICPi-induced AKI is being observed with increasing frequency in patients. In the largest retrospective study available, AKI occurred at a median time of 16 weeks (IQR 8–32) following ICPi initiation [49]. When a kidney biopsy was performed, the typical lesion associated with AKI was acute tubulointerstitial nephritis (ATIN) [49]. Thus, it is likely that nephrologists will be increasingly charged with diagnosing and managing AKI following ICPi administration [103]. Accordingly, an increasing number of case reports have described kidney complications and AKI associated with the use of ipilimumab and/or nivolumab [103,104]. The first reported cases of AKI linked to nivolumab were described in 2016 [105] and were associated with ATIN. This association raised the possibility that nivolumab therapy may release the suppression of T-cell immunity that normally permits renal tolerance to drugs known to be associated with ATIN [105,106]. In addition, PD-1 knockout mice were shown to spontaneously develop glomerulonephritis [107,108], suggesting that PD-1 inhibitor therapy may drive an autoimmune variant of interstitial nephritis. Though very little has been reported about AKI pathophysiology linked to nivolumab and ipilimumab, AKI appears as a delayed onset following the exposure to ICPi [103]. This is in stark contrast with cytotoxic-induced AKI, which is rather immediate. In addition, ICPi-induced AKI presents many features of autoimmune diseases rather than of direct drug-related nephrotoxicity, likely explaining the delayed onset of AKI in these patients. However, the underlying mechanisms of kidney injury are largely unknown (and excellently reviewed here [98]) and warrant further investigation. Nevertheless, a recent paper reported two AKI cases in patients with nivolumab treatment, characterized by the presence of karyomegalic TEC, potentially indicating TEC polyploidy. Of note, most of the enlarged tubular epithelial cells were positive for Ki-67, a cell cycle activation marker [109]. Ki-67 cannot distinguish cells undergoing mitotic or alternative cell cycles, but rather it indicates cell cycle entry [109]. This may indicate that karyomegalic TEC are polyploid cells undergoing multiple rounds of polyploidization [109,110].

Targeted agents. Molecular-targeted agents are compounds that target specific molecules involved in the growth and spread of cancer cells [111]. In respect to cytotoxic agents, targeted agents are thought to have fewer side effects and cause less harm to non-cancer cells. Epidermal growth factor receptor inhibitors (EGFR inhibitors) are used extensively to treat various cancers, such as non-small-cell lung cancer, breast, head and neck, and pancreatic cancer [112]. Epidermal growth factor receptor (EGFR) is a transmembrane protein with intrinsic tyrosine kinase activity that can be activated by several ligands, modulating cell differentiation, proliferation, and survival through the EGFR–ERK and EGFR–PI3K–Akt signaling pathways [113,114]. The blockade of EGFR may result in AKI, nephrotic syndrome, and proliferative glomerulonephritis [115,116]. The exact pathogenesis of EGFR inhibitors-associated kidney-related disorders is unclear [112]. However, it should be noted that EGFR is widely expressed in mammalian kidneys [117]. In this respect, functional analysis performed in vivo showed that treatment with an EGFR tyrosine kinase inhibitor (erlotinib, a commonly used anti-cancer agent [118]) delayed renal function recovery after AKI [114]. In contrast, EGFR activation accelerated kidney repair [114]. Indeed, proximal tubular EGFR knock-out (KO) mice showed persistent tubular cell damage in the weeks after AKI compared to wild-type mice [114]. Interestingly, no difference was detected in innate immune system activation and inflammatory cell infiltration [114]. This implies that the delayed recovery rate of EGFR-KO mice is related to a direct effect on TEC rather than a systemic effect. Recently, activation of the EGFR-PI3K-Akt pathway in response to AKI was shown to activate Yes-associated protein (YAP1), promoting kidney repair [119]. YAP1 is the main effector of the highly conserved Hippo pathway [120]. Unlike other signaling pathways, the Hippo pathway does not have dedicated receptors, but it is rather regulated by a network of upstream components [120]. This pathway appears to work as a sensor for tissue integrity, responding and adapting accordingly [120]. Importantly, YAP1 has been reported to control polyploidization in the liver through Akt signaling [121]. In the liver, YAP1 activation turns on Akt signaling, promoting S-Phase Kinase Associated Protein 2 (SPK2) acetylation, resulting in cytoplasmic retention, leading to cell polyploidy [121]. Interestingly, loss of liver kinase B1 (LKB1) in mouse hepatocytes enhances EGFR activation, which leads, in turn, to mitotic slippage and increased cell polyploidization [122]. Collectively, the importance of polyploidization [54,123] and EGFR downstream activation of YAP1 in response to AKI [114] may indicate that blocks of EGFR could profoundly affect the endogenous repair potential of the kidney, especially when administered in combination with cytotoxic agents that are notoriously nephrotoxic, exacerbating their damage.

In addition to EGFR inhibitors, targeted agents that have been shown to trigger AKI are BRAF blockers [124,125], B-cell lymphoma-2 inhibitors [126], and BCR-ABL1 and receptor tyrosine kinase inhibitor [127,128,129]. However, in most cases, the association remains vague and requires thorough investigation.

## 5. Biomarker-Guided Diagnosis

The Acute Disease Quality Initiative (ADQI) group proposed an extended definition of AKI, which includes AKI biomarkers classified as functional and damage biomarkers according to the AKI aspects, which they recapitulate. SCr level and urine output are two functional biomarkers widely employed in clinical practice, but they have several limitations [140]. Indeed, in healthy patients, the sCr levels increase only if at least 50% of the functional nephrons are lost, whereas during critical illness (i.e., in ICU patients), many confounding factors likely play a role in creatinine decrease (i.e., cirrhosis, hyperbilirubinemia, fluid overload, elderly patients, muscle wasting), making eGFR based on creatinine unreliable to correctly estimate kidney function [141,142]. These caveats limit the ability of sCr measurements to diagnose early AKI. In addition, creatinine assessment does not clarify to what extent subclinical AKI episodes contribute to shortening the kidney lifespan and CKD. Likewise, urine output can be influenced by hypovolaemia and the use of diuretics, resulting in a relatively low specificity of this parameter [143]. Cystatin C is a low molecular weight molecule produced by all epithelial cells. It is freely filtered by the glomerular filtration barrier and completely reabsorbed by proximal TEC in healthy individuals [144]. Therefore, it is detected in the urine only following tubular epithelial injury. Unlike sCr, its measurement is not confounded by acute and chronic illness, changes in diet, and decreased muscle mass, rendering it a better predictor of mortality compared to the sCr-based eGFR calculations [142,145,146,147,148].

In contrast to functional biomarkers, damage-associated biomarkers are specific to tubular injury and can potentially identify patients at higher risk of developing AKI. This is particularly relevant in clinically silent cases or in subclinical AKI, where creatinine level and urine output measurements are unreliable. In fact, early kidney damage does not often cause a relevant change in urine output or sCr, missing the diagnostic criteria of AKI. If the damage is severe or prolonged over time (i.e., progression to AKD), overcoming the renal function reserve, a GFR reduction and a subsequent alteration of sCr and urine output will be observed. However, kidney damage without any function loss also affects outcomes [149,150]. Importantly, both functional impairment (sCr level elevation and/or urine output decline) and the presence of damage biomarkers indicating structural damage are associated with a marked mortality increase in specific clinical contexts, such as those associated with critical illness [151,152,153,154]. Therefore, identifying patients at high risk of chemotherapy-associated AKI development is a challenge, and damage biomarkers offer a potential solution to guide clinicians in their therapeutic decisions to prevent AKI outcomes (Table 2) [155].

*Damage biomarkers.* Nephrotoxins contribute to approximately 30% of AKI cases in critically ill patients, and mismanagement from excessive nephrotoxic treatment coupled with unnecessary exposure is often a contributing factor [156,157]. As the use of nephrotoxic agents represents one of the few modifiable risk factors for AKI, clinicians must be able to rapidly identify patients at high risk for drug-induced kidney injury.

Several biomarkers with a different anatomical origin, kinetics, function, and timing after the insult have been identified and used for clinical and/or research purposes. These molecules are usually produced after a parenchymal lesion and released in the urine due to tubular reabsorption failure. Several neutrophil gelatinase-associated lipocalin (NGAL) isoforms are released by the kidney (thick ascending limb and collecting ducts) and by immune cells [158]. In healthy individuals, the concentration of NGAL in the urine is very low, but it increases considerably after an insult, showing high sensitivity and specificity for predicting AKI in patients with a previously normal kidney function [159,160], as well as in patients with CKD [161,162]. Several in vivo studies evaluating the NGAL response to known nephrotoxins, including aminoglycosides, amphotericin B, cisplatin, paraquat poisoning, methotrexate, nonsteroidal anti-inflammatory drugs, and vancomycin, are described [130,131,132,163,164].

NGAL measurement in cisplatin and amphotericin-associated AKI was effective in the early detection of AKI, performing better than sCr, but it was not so evident in chronic cisplatin-associated AKI [131,138].

Kidney Injury Molecule-1 (KIM-1) is a type 1 transmembrane glycoprotein that is markedly upregulated in the injured proximal tubular epithelium after ischemic injury or nephrotoxic exposure and shed into the tubular lumen [165]. KIM-1 is suggested to be a more sensitive/specific biomarker for detecting amphotericin and cisplatin-induced AKI [166]. Urinary KIM-1 and NGAL could efficiently discriminate patients with or without vancomycin-associated AKI earlier than sCr, and their combination showed fair discrimination compared with the individual biomarkers [137]. Further studies in patients undergoing platinum chemotherapeutics, urinary levels of KIM-1, NGAL, and cystatin C showed a statistically significant early increase after treatment initiation, preceding sCr rise, in AKI patients [133,167]. Accordingly, a Canadian study showed the ability of KIM-1 and NGAL to provide early AKI detection and their utility in identifying patients at risk of long-term AKI complications in a cohort of pediatric oncologic patients [130]. Following this, the US Food and Drug Administration (FDA) approved KIM-1 as a nephrotoxic biomarker for different drugs in use, resulting in several quantitative KIM-1 measurements having been developed [168,169,170]. Liver-type Fatty Acid-Binding Protein (L-FABP) is mainly produced by the liver but also by other organs, such as the kidney. L-FABP can be detected in the urine predicting AKI in patients after cardiac surgery or in critically ill patients, apparently better than NGAL [171,172]. Further studies demonstrated an additional benefit of using biomarkers (NGAL, KIM-1, L-FABP) in conjunction with the functional criteria of sCr and urine output, as their combination improves the prediction of worse outcomes [155]. Other biomarkers are represented by the lysosomal enzyme N-acetyl-b-D-glucosaminidase (NAG) and the cytosolic protein lactate dehydrogenase (LDH) [173,174]. The relationship between NAG and drug-induced kidney disease has been evaluated in several studies [135,163,175], focusing mainly on aminoglycoside and cisplatin use, demonstrating that higher NAG levels exhibited a relationship with nephrotoxicity during therapy with aminoglycosides and with a methotrexate and cisplatin combination [134,135,136]. In a proof-of-concept study, damage urinary biomarkers (KIM-1, NGAL, and NAG) provided an early identification of aminoglycoside-related proximal tubule renal toxicity, enabling treatment adjustment and the identification of infants at risk of long-term kidney impairment [163].

Other biomarkers of nephrotoxicity include gamma-glutamyl transpeptidase (GGT), Glutathione S-transferase (GST), and alanine aminopeptidase (AAP). GGT and NAG predicted AKI in critical care patients, especially in the ICU setting [176], and urinary concentrations of NAG increased in mice exposed to gentamicin or lithium [136,177]. Recently, urinary dickkopf-3 (DKK3), a stress-induced tubular epithelial-derived profibrotic glycoprotein, has been shown to predict postoperative AKI and provide information about ongoing tubulointerstitial fibrosis and short-term eGFR loss [177,178,179]. The RUBY study demonstrated that elevated urinary CCL14 predicts persistent AKI in a large heterogeneous cohort of critically ill patients with severe AKI [180]. However, there is still no evidence of the potential application of DKK3 and CCL14 in the context of chemotherapy-induced AKI.

*Cell cycle arrest biomarkers.* Unbiased screening for urinary biomarkers revealed that cell cycle arrest markers were among the top candidates capable of predicting subsequent AKI [181]. Cell cycle arrest of kidney TEC is involved in the pathogenesis of AKI [182]. As G1 cell cycle arrest due to cell stress is one of the first events during AKI, metalloproteinase inhibitor 2 (TIMP2) and insulin-like growth factor-binding protein 7 (IGFBP7) are detectable in the urine very early during AKI development [181,183]. In the Sapphire study [184], combined TIMP2 and IGFBP7 measurement demonstrated an excellent ability to predict moderate to severe AKI, and it was superior to all the other existing AKI markers, considerably improving patient risk stratification [185,186]. The FDA subsequently approved a test incorporating this marker combination (termed Nephro-Check) for clinical use. Several trials have shown that urinary TIMP2 and IGFBP7 levels predict AKI development, kidney recovery, and patient mortality [181,183,187,188]. The PrevAKI trial was the first study to investigate TIMP2 and IGFBP7 in diagnosing AKI associated with cardiac surgery [183,189]. Biomarker level ([TIMP-2]∙[IGFBP7] (0.3 ng/mL)^2^/1000) and time point of measurement (4 h after cardiopulmonary bypass) resulted in a successful predictive performance of those patients at high risk of AKI development. A similar biomarker-guided intervention was applied to prevent AKI after major surgery in the BigPAK trial [190]. The development of moderate as well as severe AKI, the incidence of sCr increase, ICU, and hospitalization length were all significantly reduced in patients whose biomarker levels were within the range of 0.3–2.0 (ng/mL)^2^/1000. This suggests that patients with higher biomarker levels may have suffered an extended period of kidney stress, resulting in a progression to AKI and AKD. Early biomarker-based prediction of AKI followed by implementation of KDIGO (Kidney Disease: Improving Global Outcomes) care bundle reduced AKI severity [190].

**Figure 3:**
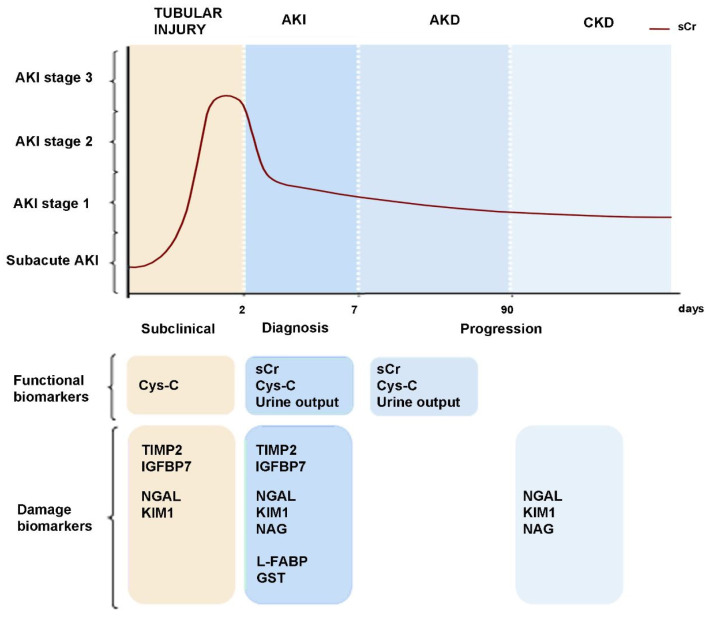
Biomarkers’ detection in AKI, AKD, and CKD. Among the currently used biomarkers, only a few of them are detected early during AKI progression, before a rise in the serum creatinine (sCr) is present. These markers indicate early tubular injury or subclinical acute kidney injury (AKI). AKI itself is recognized by both functional and damage biomarkers, whereas the stages of AKI are defined by the extent of kidney function impairment represented by sCr rise. AKI accompanied by prolonged tubular damage is defined as acute kidney disease (AKD). When the injury is extended and irreversible, and kidney function cannot be restored, it leads to chronic kidney disease (CKD). Abbreviations: Cys-C, cystatin C; IGFBP-7; insulin-like growth factor-binding protein 7; TIMP-2; metalloproteinase inhibitor 2; GST: Glutathione S-transferase; NAG: N-Acetyl-Beta-D-Glucosaminidase; NGAL: Neutrophil Gelatinase-Associated Lipocalin; KIM-1: Kidney Injury Molecule-1; L-FABP: Liver-type Fatty Acid-Binding Protein.

Based on the Nephro-Check test results, in both PrevAKI and BigPAK studies, patients with a higher risk of AKI had benefited from the decision to avoid nephrotoxic treatment [189,190]. Thus, it is conceivable that implementing a biomarker-based approach with the detection of the high-risk population might be beneficial for preventing AKI. These biomarkers can also be used to predict adverse long-term outcomes because their early measurement in the setting of critical illness may identify patients with AKI at increased risk of death or KRT in the following months [187]. Moreover, the best results can be achieved by combining different biomarkers. High KIM-1, NGAL, and [TIMP-2]∙[IGFBP7] values identified patients with vancomycin-associated AKI earlier than sCr [137,139]. A drug combination that has gained recent attention for an additive risk of nephrotoxicity is vancomycin plus piperacillin–tazobactam. In order to establish whether kidney injury associated with this combination is a valid clinical concern, [TIMP-2]∙[IGFBP7] have been employed. Patients treated with the combination therapy showed higher levels of [TIMP-2]∙[IGFBP7] in comparison to those treated with vancomycin monotherapy, associated with increased long-term adverse outcomes [191]. Collectively, this evidence suggests the benefit of damage biomarker measurement in identifying nephrotoxic AKI early (Figure 3). Practical considerations for the implementation of these biomarkers for predicting and detecting chemotherapy-induced kidney injury need to be evaluated. In particular, a better understanding of the appropriate concentration for each biomarker for each nephrotoxic drug or drug class that increases the risk for drug-induced kidney injury needs to be developed.

## 6. Management

*General measures.* Given that no specific evidence is available to suggest that AKI in cancer patients should be managed differently from other causes of AKI, strategies based on KDIGO are appropriate for risk- and stage-based prevention and management of AKI [140]. Cancer patients are particularly at risk for infection and sepsis [18,192]. Thus, early detection and management of sepsis, including source control of the infection (e.g., removal of tunneled central venous catheter systems) and optimized antibiotic use based on the pharmacokinetics and pharmacodynamics changes observed in AKI are essential, particularly in patients with neutropenia [18,193]. A review of patients’ charts to ascertain the cumulative exposure to chemo- and immunotherapeutic agents and other medications is important to assess the risk of nephrotoxicity and other less common therapy-associated injuries (e.g., thrombotic microangiopathy, tubulointerstitial nephritis, glomerular diseases, and intratubular obstruction from medications) must also be considered [44]. Notably, the risk of nephrotoxicity increases from cumulative exposure to chemotherapeutic agents and other medications [194,195]. The risk of AKI increases with the number of nephrotoxic drugs used, and all potentially nephrotoxic agents that can be stopped should be discontinued [146,196]. Indispensable agents should only be used as long as needed and only at required doses. Careful monitoring of drug concentrations is also mandatory (for example, vancomycin) [44].

Patients with chemotherapy-induced AKI may present with symptoms and signs resulting directly from diminished kidney function. These typically include edema, hypertension, decreased urine output, or anuria in severe AKI [1]. However, many patients do not show any symptoms, and the only sign of diminished kidney function may be an increase in creatinine detected by laboratory tests without an overt AKI [1]; otherwise signs and symptoms are indistinguishable from AKI from other etiologies. SCr remains the only laboratory value used in operative definitions for AKI and the biomarker most used in clinical practice. All subsequent evaluations are directed at determining the underlying cause of AKI to achieve prompt and adequate management. For all patients, the timing of onset often suggests the underlying etiology, albeit sCr concentration should be measured frequently, a goal hardly achievable unless the patient is admitted to the hospital. Careful attention should be given to volume status to avoid hypovolemia, as patients may initially present with relative volume depletion due to fever and gastrointestinal losses as volume resuscitation is rarely performed [197]. Volume management and hemodynamic monitoring are also required at all stages of AKI. Avoiding hyperglycemia is also essential because the filtered glucose increases tubular reabsorption workload and oxidative stress, a process that sensitizes the kidney tubule to injury [198]. Implementation of the ‘KDIGO bundle’—consisting of optimizing volume status and hemodynamics, avoiding nephrotoxic drugs, and preventing hyperglycemia in patients at high risk of AKI as identified by biomarkers—can prevent AKI [189].

*Kidney replacement therapies.* When the severity of AKI necessitates KRT, the jugular veins should be considered as the preferred insertion sites for dialysis catheters. The catheter exit site and anchoring remain visible, and these sites confer a lower risk of infection and thrombosis [199]. Initiation and continuation of dialysis in the cancer patient with AKI should be based on the general clinical condition and overall life expectancy and the personal patient expectations on quality of life after eventual recovery [200]. Hypophosphatemia is common in malnourished cancer patients and those on prolonged continuous KRT and may need to be corrected with supplements to prevent hypophosphatemia-associated complications [201]. Intradialytic seizures may occur in cancer patients on maintenance anticonvulsant therapy due to dialytic removal of the drug, and higher post-dialysis doses may be required to maintain therapeutic serum concentration [202]. Cancer patients are at risk of malnutrition due to various factors, such as prolonged immobilization, catabolic changes, and reduced food intake. Therefore, the current consensus recommendations for the nutritional management of critically ill patients with cancer should be followed [203]. Finally, kidney transplantation is not a valid KRT in the critically ill patient perspective.

*AKI to CKD transition.* Limiting progression from AKI and AKD to CKD is a crucial issue in chemotherapy-exposed patients [204]. Cancer is strictly linked to AKI and CKD, and the presence of CKD markedly reduces cancer patients’ survival [205,206,207]. Patients with risk factors for CKD (i.e., diabetes, hypertension, obesity, low nephron endowment, and many others) on the verge of receiving chemotherapeutics should be trained adequately about possible CKD onset and progression [2]. All the risk factors for CKD mentioned above should be tightly controlled whenever possible: patients should implement a healthy diet and physical activity, and anemia, high blood pressure, dyslipidemia, and diabetes should be pharmacologically controlled when conservative measures prove to be insufficient [2]. It would be advisable to stop medications that may increase the risk for nephrotoxicity, namely non-steroidal anti-inflammatory drugs, whenever clinically feasible [189]. Exposure to iodinated contrast should be limited, too [189]. Concerning cancer patients, all lifestyle modifications and new drugs implementation should always be collectively discussed with patients and oncologists. In this setting, nephron overload, the structural adaptations that promote accelerated loss of kidney epithelia in nephrons challenged by hemodynamic and metabolic overload, represents a typical driver of CKD progression and a therapeutic target [208]. Currently, the renin–angiotensin–aldosterone system and SGLT2 (Sodium–glucose Cotransporter-2) inhibitors represent the most effective drugs to slow CKD progression [209,210,211]. Importantly, there is no evidence to date linking SGLT2 inhibitors and an augmented risk of cancer [212].

**Figure 4:**
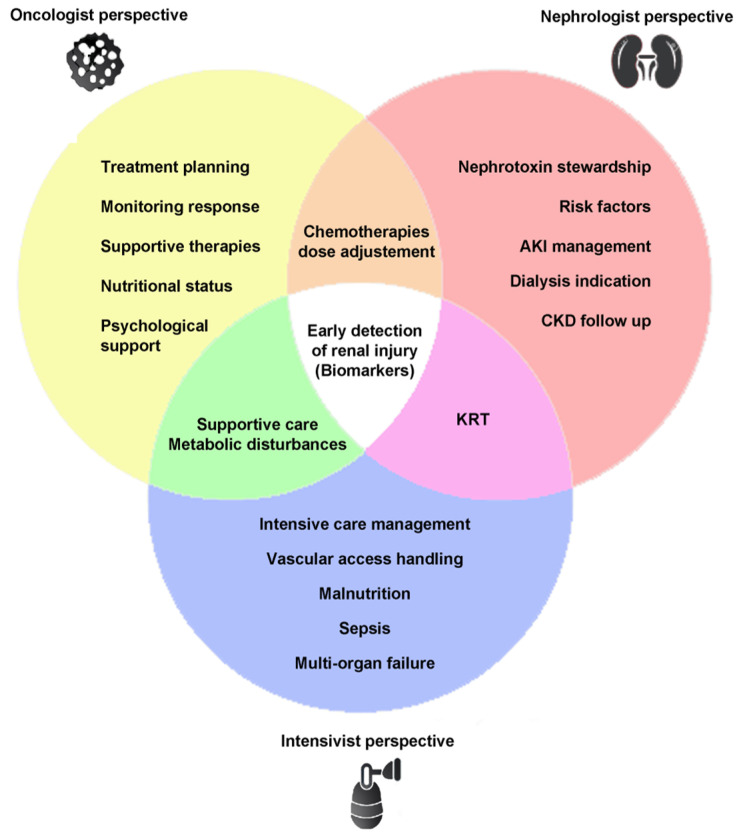
The ideal management of cancer patients with nephrotoxic-AKI. Venn diagram representing the aspects to be considered when treating patients for early acute kidney injury (AKI) recognition and effective oncologic patient management. Abbreviations: KRT: Kidney Replacement Therapy; CKD: Chronic Kidney Disease.

*Future directions.* Improvement in AKI diagnosis and treatment remains a significant unmet medical need. Given AKI is a global health problem, there is an urgent need to train health workers to identify patients at significant risk of kidney disease development and subsequent progression to AKD or CKD. An active and effective proposal should span from health-system surveillance methods to clinical interventions. This should be done by: (1) promoting a stronger collaboration between nephrologists, intensive care specialists, and oncologists; (2) preventing or at least limiting drug-associated AKI through nephrotoxin stewardship, and (3) implementing novel biomarkers aimed at a proper patient classification [213] (Figure 4). The ideal biomarker “for” AKI should be (1) sensitive, it should work as an early predictor of AKI and then be altered following injury in a period of minutes or hours; (2) AKI specific by providing clues regarding the underlying etiology; (3) serve as a prognostic factor; (4) predict the need for KRT; (5) be cost-effective and highly reproducible.

Hence, it is essential to use the best available and novel biomarkers to recognize initial AKI phases and apply protective measures and risk mitigation to avoid worsening of the condition. Finally, even when AKI has fully developed, identifying patients who might progress to AKD or even CKD is important [214]. In these patients, specific biomarkers may help plan the allocation of resources.

## 7. Conclusions

The true incidence of AKI-associated nephrotoxicity is unknown, and little progress has been made in its treatment and prevention. This is likely due to a significant gap in our knowledge of kidney response mechanisms to AKI. We recently demonstrated that polyploidy of TEC represents a previously unrecognized mechanism of response to kidney damage. These polyploid TEC were found arrested in G1 [54,215], suggesting an intriguing parallel with the Nephro-Check assay that correlates damage biomarkers of cell cycle arrest with the likelihood of developing AKI. Identifying novel response mechanisms may help advance and implement kidney injury markers to improve AKI diagnosis. Increasing evidence suggests that a biomarker-based approach could be promising for identifying patients at high risk of developing AKI. This is essential to prevent and ameliorate the occurrence of AKI and chemotherapy associated-AKI and to assist in the early management of patients with chemotherapy-associated-AKI. Despite the rapid evolution of research in this field, the diagnostic performance of these renal biomarkers has demonstrated a number of limitations and highlighted substantial gaps in our knowledge, which likely reflect the absence of accepted standard criteria [140,216]. Some damage biomarkers perform differently based on the patient population studied, the presence of pre-existing CKD, and whether a clinical risk model for high-risk individuals was used before applying the biomarkers. Thus, the existing evidence for using biomarkers to monitor the effects of medications and cope with their management has yet to consider the complex list of confounders that could affect their diagnostic performance. Accordingly, recommendations from the 23rd ADQI consensus conference suggest that combining AKI definitions based on sCr and urinary output with kidney injury biomarkers would improve the precision of AKI course prognostication [146].

In conclusion, we have only begun to understand the potential advantages of integrating damage biomarkers into daily clinical practice, and future studies are required to appreciate the impact on patient care correctly. Further research is needed to clarify whether detecting damage biomarkers without any changes in urine output or sCr is associated with worsened kidney and patient outcomes.

## Figures and Tables

**Table 1:** Definitions.

Term	Definition	Available Classification	Reference
Acute Kidney Injury (AKI)	Acute kidney injury (AKI) describes a sudden loss of kidney function that is determined on the basis of increased sCr levels (a marker of kidney excretory function) and reduced urinary output (oliguria) (a quantitative marker of urine production) and is limited to a duration of 7 days.	RIFLE (2004) AKIN (2007) KDIGO (2012)	[1] [24] [25] [26]
Acute Kidney Disease and Disorders (AKD)	A variety of functional kidney conditions that can range from mild and self-limiting to severe and persistent. AKD persisting for >3 months is referred to as CKD.		[1]
Nephrotoxicity	The damage of kidneys by exogenous or endogenous toxicants.		[27]
Chronic Kidney Disease (CKD)	CKD is a syndrome defined as persistent alterations in kidney structure, function, or both with implications for the health of the individual.	KDIGO (2013)	[2] [28]

sCr: serum Creatinine; RIFLE: Risk, Injury, Failure, Loss, and End-Stage Renal Disease; AKIN: Acute Kidney Injury Network; KDIGO: Kidney Disease Improving Global Outcomes.

**Table 2:** Chemotherapy induced-AKI, mechanisms of nephrotoxicity and associated biomarkers.

Drug	Class of Antineoplastic Drug	Kidney Associated Clinical Features	Mechanism of Nephrotoxicity	Biomarkers	References
Cisplatin	Platinum agents	AKI Hypomagnesemia	Oxidative stress and inflammation, DNA damage-induced apoptosis and polyploidy	NAG NGAL KIM-1	[56,130,131,132,133]
Ifosfamide	Alkylating agents	AKI Nephrogenic diabetes insipidus Fanconi syndrome dRTA	Oxidative stress, DNA damage, and karyomegalic nephropathy		[75,78,85,86,87,88]
Vancomycin, Gentamicin, and Amphotericin B	Anti-infectives drugs	AKI	Not well understood, DNA damage and oxidative stress on proximal tubular cells	NAG NGAL, KIM-1 [TIMP-2][IGFBP7]	[89,132,134,135,136,137,138,139]
Erlotinib	EGFR inhibitors	AKI Nephrotic syndrome and proliferative glomerulonephritis	Not completely understood, block of YAP-1 impairs kidney repair		[114,118,119]
Nivolumab	Immune checkpoint inhibitors	AKI delayed onset	Largely unknown, finding of TEC polyploidy		[103,109,110]

AKI: Acute Kidney Injury; IGFBP-7, Insulin-Like Growth Factor-Binding Protein 7; TIMP-2, Tissue Inhibitor of Metalloproteinase 2; NAG: N-Acetyl-Beta-D-Glucosaminidase; NGAL: Neutrophil Gelatinase-Associated Lipocalin; KIM-1: Kidney Injury Molecule-1; EGFR: Epithelial Growth Factor Receptor; TEC: Tubular Epithelial Cells; dRTA: distal Renal Tubular Acidosis; YAP-1: Yes-Associated Protein 1.

## Data Availability

Not applicable.
